# Hope, passion and perseverance: experiences of internationally educated nurses pursuing nursing authorisation in Norway–a qualitative study

**DOI:** 10.1186/s12912-024-02297-x

**Published:** 2024-09-11

**Authors:** Daniela Lillekroken, Line Nortvedt

**Affiliations:** Department of Nursing and Health Promotion, Oslo Metropolitan University, PB 4 St. Olavs Plass, Oslo, N-0130 Norway

**Keywords:** Bridging programmes, Digital teaching, Individual interviews, Internationally educated nurses, Nursing education, Thematic analysis

## Abstract

**Background:**

The global nursing shortage is driving nursing professionals to migrate from their home countries to other regions of the world, leading to increased diversity in healthcare settings and nursing education across Europe. Although research on the experiences of internationally educated nurses has gained more attention in Norway, a substantial gap remains in understanding the challenges these nurses face when participating in bridging programs and seeking authorisation as registered nurses in the host country.

**Methods:**

The aim of the present study is to gain knowledge about the experiences of students in a digitised bridging program for nurses, related to being a nurse educated outside the European Union and a student with a Norwegian as a second language and migrant/refugee background. Oslo Metropolitan University initiated a decentralised education programme in 2021, offering a bridging programme for individuals with a nursing education from countries outside the European Union. This programme was conducted in a decentralised, gathering-based, and predominantly digitised format. The study has a qualitative descriptive design and includes a purposive sample of eight former nursing students enrolled in the programme. Data were collected through individual semistructured interviews conducted between November and December 2023. The data were analysed by employing thematic analysis. The study is reported in accordance with the COnsolidated criteria for REporting Qualitative research (COREQ).

**Results:**

Analyses revealed a main theme—the participants’ ability to persist in their goal over the long term, maintaining their interest, overcoming challenges, working hard and finishing tasks rather than giving up. This theme is supported by three themes: (i) ‘Navigating bureaucratic challenges – The struggle with authorisation and overwhelming requirements, (ii) ‘An emotional journey – The ups and downs of participating in the program’, and (iii) ‘Achieving recognition – The journey to authorisation and professional confidence’.

**Conclusions:**

The study underscores the numerous challenges encountered by internationally educated nurses seeking registered nurse’ recognition in Norway. Despite these challenges, the participants displayed remarkable hope, passion and perseverance, remaining committed to their goal of becoming registered nurses in Norway.

**Supplementary Information:**

The online version contains supplementary material available at 10.1186/s12912-024-02297-x.

## Background

Nurses are an important professional group for the healthcare system, and according to the World Health Organization [1], they comprise approximately 50% of the world’s healthcare personnel. Prior to the pandemic, the international migration of nurses was already significant and, on the rise [2]. The Organisation for Economic Cooperation and Development’s (OECD) analysis revealed that, in 2019, over 550,000 nurses trained abroad were employed across 36 OECD member countries. This marked a notable increase from the 460,000 reported in 2011 [3].

There is an escalating gap between the demand for nurses and the available supply; therefore, Western countries have responded to their nursing deficits by recruiting internationally educated nurses (IENs) [4]. Results from a report leaded by International Council of Nurses (ICN) [5], show that the global healthcare landscape is facing a critical shortage of nursing professionals because of the impacts and aftermath of the pandemic, the need for rebuilding, worsening rates of chronic diseases and the ageing population in numerous countries. Meanwhile, another ICN report [6] demonstrates that the supply of nurses is dwindling because of burnout caused by the pandemic, resulting in outflows, as well as underlying demographic shifts such as ageing of the workforce. Figures from Economics Norway state that, in 2018, there was a deficit of 2,500 authorised nurses in Norway and that this number will increase tenfold by 2035 [7].

IENs often face challenges that can lead to poor professional development and a devaluation of their competence in host countries [8]. Some of these challenges are related to differences in nursing, adaptation to their new nursing roles and workplace culture and the evolution of the self [9]. Others are related to cultural differences, leading to feelings of being outsiders [10] and discrimination [11]. The expectations IENs had prior to migration may not be met and that in turn could have a negative impact on the integration process and their retention [12]. These challenges show the need for good transition programmes to ensure that nurses reach their full career potential as professional nurses [13].

While several studies [14–18] have focused on the integration strategies and factors that underpin a successful integration of IENs in the host countries, and IENs experiences working in the host country [10, 19, 20], limited research has been conducted on the relationships between nursing education, digitisation programs and IENs who attend bridging programs.

Two Swedish studies [21, 22] involving qualitative interviews with individuals studying to obtain Swedish nursing authorisation show how the students mainly found bridging programmes challenging, but also rewarding. While Hadziabdic et al. [21] explored the experiences of nurses educated outside the European countries, in the Swedish bridging program and its role in their integration into the nursing profession, Högstedt et al. [22] have explored complementary education outside English-speaking countries. Both studies described the challenges of studying with a new language in a new healthcare system. Students experienced being underestimated and misunderstood, but at the same time, they experienced joy and pride when they felt more confident practicing nursing in their new home country. However, achieving goals was also dependent on one’s personal characteristics such as strong will and knowledge, professional experience, and language proficiency, as well as development opportunities that were available such as formal and informal support [21]. The informants highlighted the importance of educators and mentors being supportive and understanding of their background and language challenges, but some noted that it could be difficult to get to know both educators and fellow students because the teaching was conducted online [22].

Challenges encountered by IENs while completing bridging programs and securing jobs have been highlighted in several studies. A Finnish case study with 20 participants revealed that the main motivation for Filipino nurses working in Finland was to gain access to Europe, save money and send part of their earnings home [23]. These nurses hoped to have their four-year Filipino nursing education recognised in Finland. However, Finnish employers recruited these professionally skilled and experienced nurses from the Philippines and merely employed them as assistants [23]. Another study conducted in Finland [24] involving 10 Filipino nurses concluded with a recommendation to recognise prior education and clinical experience, which could facilitate the transition to becoming a RN in Finland for IENs. Although bridging programmes are available in Finland, the lack of a clear educational model and recognition of foreign nursing qualifications has resulted in deskilling, preventing IENs from practicing [24].

Filipino nurses who have migrated to Norway to work experienced unfairness and difficulty because they did not receive authorisation, even though they considered themselves well qualified [25]. Many had good grades from their home country and felt exploited, underpaid and devalued in Norway. Nursing leaders play therefore a crucial role in including and ensuring that IENs have good working conditions [26]. Moreover, it is recommended that IENs should be supported during the transitional period and guided by Norwegian colleagues [26]. This is also supported by a study conducted in Singapore [27], which suggests the use of mentorship programmes during IENs transition from education programmes into clinical setting. Additionally, another study analysing students enrolled in a one-year bridging programme for IENs in Oslo, Norway, reveals how students experienced professional degradation postmigration [28]. The study highlights the importance of IENs’ resilience displayed in response to such challenges. Their resilience is bolstered when they anticipate the situation as being temporary, have clear routes for skill enhancement and can access social and economic support for requalification. Furthermore, a strong professional identity and confidence in their abilities to contribute to Norwegian society serve as vital sources of resilience [29].

Establishing bridging programmes offers IENs a structured pathway for requalification, authorisation and eventual employment, fostering hope for professional reintegration [28]. Bridging programs have recently been introduced into the Norwegian education system. In 2017, at the request of the Ministry of Education and Research, Oslo Metropolitan University (OsloMet) launched a 60 ECTS bridging program for nurses trained outside the European Union/European Economic Area (EU/EEA). This program, known as SKOMP, is designed to equip refugees and migrant workers with the necessary qualifications to enter the Norwegian labour market through supplementary education. According to the Norwegian Directorate of Health, the minimum requirements for nurses educated outside the EU/EEA include completing language requirements (B2 level^1^), courses in drug handling, and a skills test. However, to be enrolled in the SKOMP program at Oslo Metropolitan University [30], applicants should meet the following requirements:

- Have completed nursing education from a country outside the EU/EEA.
- Meet proficiency requirements in Norwegian (Level B) and English.
- Have received a decision from the Norwegian Directorate of Health stating that additional education is required to achieve equivalent nursing qualifications for obtaining authorisation as a nurse in Norway (the decision must be dated after June 1, 2015).
- Applicants with a refugee background should upload a letter from the Norwegian Directorate of Immigration (UDI) with a decision on residence permit where the applicant has the status of a refugee.

In her study, Gotehus [31] focused on acts of resilience and adaptation to explore how Filipino migrant nurses navigate and overcome the barriers encountered while seeking to enter the Norwegian labour market and integrate into Norwegian society. Another study [32], examined Filipino and Polish migrant nurses in Norway, revealing that, despite varying structural conditions, both groups encountered challenges to their professional identities, which were characterised by feelings of decompetence. In this context, decompetence can be defined as a subjective and context-dependent process in which the nurses’ education and professional expertise are underutilised or not applied meaningfully [32]. Their study revealed that IENs employed several coping strategies to confront, manage or even leverage the realities of working within the Norwegian healthcare system. As outlined earlier, numerous studies have explored the challenges IENs face from their arrival in the host country until they attain authorisation. However, while many studies have focused on IENs’ experiences with the authorisation process and working abroad, none have specifically examined their experiences with digitised nursing education programs for obtaining authorisation. Therefore, further research in this area is needed.

## Methods

### The aim of the study

The aim of the present study is to gain knowledge about the experiences of students in DIGISKOMP-D, related to being a nurse educated outside the EU/EEA and a student with a Norwegian as a second language (N2) and migrant/refugee background.

### Research design

The current study has a qualitative descriptive design and adheres to the qualitative description described by Sandelowski [33]. The choice of employing a qualitative descriptive research method is grounded in researchers’ wish to facilitate the inductive discovery of patterns and themes around a life event [34]; here, IEN students’ experiences with attending the DIGISKOMP-D programme. As such, a qualitative descriptive study design has been employed to answer the following research question: What are the students’ experiences with a bridging programme, conducted in a decentralised, gathering-based, and predominantly digitised format (DIGISKOMP-D), related to being an IEN and a student with Norwegian as a second language?

The Consolidated Criteria for Reporting Qualitative Research (COREQ) [35] has been used to report the findings of the current study (Supplementary File 1).

### Setting of the study

In Norway, the Ministry of Education and Research has developed a one-year bridging programme for individuals with refugee backgrounds who completed their nursing education from countries outside the EU/EEA. This education programme started in Oslo in 2017. The goal of the Oslo-based full-time education, called Supplementary Education for Nurses educated outside the EU (SKOMP), is for nurses with a nurse education background to complement their education from their country, so that they can obtain Norwegian authorisation as RNs [30].

SKOMP spans two terms. In the first term, students cover the scientific and social foundations of nursing (15 ECTS), pharmaceuticals (2 ECTS), and clinical studies in geriatrics (13 ECTS). The second term includes bachelor assignments (15 ECTS), clinical studies in psychiatry (15 ECTS), and a skills test (0 ECTS).

In August 2021, a supplementary, decentralised education (SKOMPD) started, being offered to individuals residing in Nordland County, and was conducted as a decentralised, gathering-based and mainly digitised offer, running over three semesters [36]. The present study was conducted within the context of supplementary, decentralised education (SKOMPD) for the period 2021–2022, a bridging programme, conducted in a decentralised, gathering-based and predominantly digitised format (DIGISKOMP-D).

### Sample and recruitment

The sample comprised IENs, specifically students who attended the DIGISKOMP-D programme at Oslo Metropolitan University during 2021–2022. One of the researchers was the leader of the programme and she knew the participants prior study commencement. The participants were purposely recruited. Out of the 19 IEN enrolled in the programme, eight individuals agreed to participate. To recruit participants, one of the researchers (LN) sent emails containing information about the study. Several reminders were sent.

The only inclusion criterion was that the participants needed to be IENs who had taken part in the programme. At the time of the interview, all participants had obtained authorisation as registered nurses (RNs) and were employed, working in long-term care facilities, home-based care or similar healthcare services. The sample consisted of four females and four males aged between 32 and 41 years. Seven individuals were from the Philippines, and one was from an East African country. All of them graduated as nurses in their home countries in the 2000s. An overview of the participants’ profiles is presented in Table 1.

**Table 1:** Participants’ demographic data

Participant number	Gender	Age (years)	Year of nursing education completion in their home country	Year of arrival in Norway	Year of receiving authorisation as RN in Norway
P1	Female	32	2011	2012	2022
P2	Female	36	2007	2011	2022
P3	Male	37	2007	2011	2023
P4	Female	38	2006	2015	2022
P5	Male	38	2006	2012	2022
P6	Male	41	2004	2014	2022
P7	Male	37	2008	2012	2022
P8	Female	37	2007	2010	2023

### Data collection method

Prior to the interviews, a semistructured interview guide was developed (Table 2). Both researchers—the authors of the manuscript—interviewed the participants (four participants each). At the time of the study, both researchers were employed as nurse educators at the university.

**Table 2:** Interview guide

Themes	Main questions and follow-up questions
Introduction	Information about the researchers, project objectives, interview process, informed consent details, participant anonymity, interview duration, and audio recording procedures.
Personal information about the participants	1. Can you please introduce yourself? 2. Where did you receive your education? 3. When did you complete your nursing education? 4. What is your age? 5. Can you please tell me about your family, both in your home country and in Norway.
Migration and work experience	1. Can you please describe why did you choose to migrate to Norway? 2. When did you arrive in Norway? 3. When did you obtain nursing authorization in Norway? 4. Can you share any significant experiences during your migration journey? 5. Have you worked in your home country? If so, where and for how long?
Educational background	1. Can you please describe what is your educational nursing background? 2. Where did you study nursing? 3. What was the duration of your education?
Motivation for DIGISKOMP-D	1. Can you please describe your motivation to enrol in the bridging part-time education? 2. Was it a decision influenced by the Norwegian Directorate of Health, personal aspirations, recommendations, or other factors?
Student experience	1. Can you please describe your experience as a student in the bridging programme? 2. Can you share some positive and negative aspects of your experience?
Facilitators, barriers, and teaching experiences	1. What aspects of supplementary education aided your progress compared to full-time education? 2. What challenges did you encounter during your studies? 3. Describe your experiences with teaching methods and educators.
Support systems	1. Who were the individuals or support networks that positively impacted your education? 2. What challenges, such as family obligations or financial constraints, affected your studies?
Motivations and resources	1. What factors were crucial for you to pursue your studies? 2. Please describe any positive factors that facilitated your educational journey. 3. What are your significant strengths or resources that contributed to your success?
Challenges as a migrant/refugee	1. Can you please discuss challenges related to being a migrant/refugee that affected your education? 2. What difficulties did you face while learning the Norwegian language as a student and as a registered nurse in Norway? 3. What challenges do you encounter in the nursing role in Norway?
Personal and external expectations	1. Please describe your self-imposed expectations regarding education. 2. How did external factors influence your educational expectations?
Authorisation and job market	1. Did you apply for nursing authorisation in Norway? 2. Can you please share your experiences with job searching, including job types and satisfaction. 3. Can you compare your experience of working as a nurse to that of a health care assistant. 4. Did employers consider your supplementary education (DIGISKOMP-D) during the hiring process?
Value and relevance of the bridging programme	1. How do you perceive the value of supplementary education? 2. Please describe how supplementary education has been useful or not useful in your current work. 3. How relevant is supplementary education in obtaining your desired job?
Future aspirations	1. Can you please share your thoughts on further education opportunities. 2. Have you considered changing jobs, or do you have thoughts about it?
Conclusion	Do you have any final thoughts or comments before we conclude the interview? Thank you for sharing your experiences!

The interviews began by openly informing the participants about the aim of the study and their possibility to withdraw from the study. Further, the researchers asked the participants to provide information about their age, education history and the details of their arrival in Norway, along with their process of receiving authorisation as RNs. The interviews were conducted via Zoom™, which is an effective mechanism for collecting qualitative data within a healthcare context because of its low-cost profile [37]. Using Zoom allowed the researchers to easily arrange the interviews with the participants at mutually convenient dates and times. The interviews took place between November and December 2023. All interviews and associated consent were recorded using the audio system integrated into Zoom. The interviews were then securely stored on a password-protected PC.

The interviews lasted between 26 and 65 min and were conducted by experienced female academics. During the interviews, the participants were in their own homes and alone in the room while the researchers were at their office at their university. The interviews were conducted during the daytime when the participants had free time. The interviews were carried out in Norwegian because it was the language that the IENs used at work. One of the criteria for enrolment in the DIGISKOMP-D programme is the ability to speak and understand the Norwegian language at the B2 level. Hence, all participants communicated in Norwegian with clarity and understanding, eliminating the need for a translator when conducting the interviews. Data collection ceased when the researchers considered that no new information had emerged [38], in this case after eight interviews. All the interviews were transcribed verbatim by both researchers, each of whom transcribed four interviews.

### Data analysis

The transcripts of the interviews generated 98 A4 pages with 1.5 line spacing and Times New Roman font size. To analyse the data, a reflexive thematic analysis [39], defined as a ‘method for developing, analysing and interpreting patterns across a qualitative dataset’ (p. 4) was used. The researchers read all eight transcripts, individually analysed their respective four interviews and subsequently convened to corroborate their initial impressions regarding the potential insights gleaned from the data.

Thematic analysis involves a six-step process that places researchers at the centre of interpreting and deriving meaning from the data, here emphasising ongoing reflexivity. Initially, both researchers independently conducted the first three steps (*familiarising with data*, *generating initial codes and searching for themes*). Subsequently, they collaboratively approached step 4 (*reviewing the themes*) during a joint meeting. For step 5 (*defining and naming the themes*), the researchers engaged in discussions and reached a consensus on the names of the themes. In the final step (*producing the report*), the researchers selected vivid and compelling extracts that linked the analysis back to the research question and literature, resulting in a comprehensive analysis report. After this last step, we identified one main theme and three themes, each supported by several subthemes, as outlined in Table 3. No software programme was used to analyse the data.

**Table 3:** Coding tree – main theme, themes, and sub-themes

Sub-themes	Themes	Main theme
Challenges with finding a job, and getting authorisation	Navigating multifaced challenges – the struggle to meet the requirements	The participants’ ability to persist in their goal over the long term, maintaining their interest, overcoming challenges, working hard, and finishing tasks rather than giving up.
Challenges with understanding, writing, and speaking Norwegian		
Challenges/ambiguities with the startup of the program		
Challenges and possibilities with a digital form of learning		
Economic challenges		
A roller coaster of feelings	An emotional journey - The ups and downs of participating in the program	
Expressing gratitude of being enrolled and attending the DIGISKOMP-D - program		
Confident, independent, and responsible	Achieving recognition - The journey to authorisation and professional confidence	
Being acknowledged for experience and knowledge		

### Rigour and reflexivity

A reflexive thematic analysis is a subjective approach [40]. Therefore, to ensure rigour and trustworthiness, the researchers reported their theoretical underpinnings, assumptions, values, experiences and skills as nurse educators, along with any biases that could influence data interpretation. Handwritten notes were utilised during the analysis process to reflect on the development of the analysis and the researchers’ own positions on the topic. Additionally, the present study includes detailed descriptions of the context, participants and methods for data collection and analysis.

The analysis was enhanced by researcher triangulation as both researchers discussed their respective codebooks. Because the data coding was conducted inductively, the codebooks initially differed in various ways; therefore, each researcher posed critical questions and offered suggestions to each another’s data coding. Through rigorous and reflexive comparison, the researchers developed a group codebook that they mutually agreed upon. In conducting and reporting the study, the quality guidance for reflexive thematic analysis [40] and the COREQ [35] were adhered to.

### Ethical perspectives

The project obtained ethical approval from the Norwegian Agency for Shared Services in Education and Research (Sikt, Ref. no. 912595) and by the leader of the Department of Nursing and Health Promotion at Oslo Metropolitan University. The study was conducted in accordance with the Helsinki Declaration [41], including informed consent, consequences and confidentiality, and with Oslo Metropolitan University’s guidelines and regulations for data research storage. Data were kept confidential and used only for this research purpose.

All participants were provided with both verbal and written information about the study, and written informed consent was obtained from each participant before conducting the interviews. The participants were explicitly informed that there would be no consequences for their participation and that no financial or other benefits were provided.

The participants were assured that withdrawing from the study at any point for any reason would not have negative repercussions on their further education or employment. This provided the participants with additional opportunities to either assent to or withdraw from the study. None of the participants chose to withdraw.

Confidentiality was maintained by assigning the participants a number, and the findings were reported cautiously to prevent potential identification through specific events unique to individual participants. After the interviews were transcribed, the Zoom recordings were deleted. The interview transcripts are securely stored in a locked cabinet to which only the researchers have access.

## Results

The analysis revealed one main theme: the participants’ ability to persist in their goal over the long term, maintaining their interest, overcoming challenges, working hard and finishing tasks rather than giving up. This main theme is supported by three themes: (i) ‘Navigating bureaucratic challenges – The struggle with authorisation and overwhelming requirements, (ii) ‘An emotional journey – The ups and downs of participating in the program’ and (iii) ‘Achieving recognition – The journey to authorisation and professional confidence’.

### Navigating bureaucratic challenges - the struggle with authorisation and overwhelming requirements

This theme reflects the participants’ experiences with multiple attempts at gaining authorisation, the significant challenges they face, and the frustration caused by numerous and burdensome requirements to practice nursing in Norway. As several participants revealed, they experienced frustration upon knowing that some IENs have previously received authorisation within two days after applying, and they struggled with the severe new requirements imposed by the Norwegian Directorate of Health:

Authorisation? Three attempts with the Norwegian Directorate of Health … There are many challenges … and the numerous requirements can be burdensome to the point where one just becomes frustrated …. (P7)

Some even admitted that, because of its severe requirements, the authorisation process seemed almost impossible to navigate. Furthermore, in addition to navigating the authorisation process, they also faced challenges in finding employment as healthcare workers, one of the several requirements for recognition as an RN: ‘I had only four months on me to find me a job to be able to live in Norway’. (P2)

Although the participants were frustrated with the incomprehensible extended duration of the authorisation process, they recognised the necessity of meeting the B2-level Norwegian language requirement. Some had attended Norwegian language courses in the Philippines before coming to Norway, but they admitted that these courses did not significantly contribute to achieving fluency in Norwegian. Other participants worked for many years as healthcare workers, but they still found it challenging to understand the language and realised that what they had studied was quite different from what was needed to effectively practice nursing or attend the programme in Norwegian to become an RN:

And for me, having worked in Norway for more than 10 years … it wasn’t easy … when you attend Norwegian schools, it’s not the same as it is in the Philippines because there, we use English, and here, we use Norwegian, and the [medical] terminology is often a bit challenging. (P3)

The participants also expressed frustration with the ambiguous information regarding starting the programme, as one of the participants stated:

So, it was in 2021 … and then, it was a stop, but then, it turned out that it didn’t happen after all … And then I saw on the internet that it’s starting again [the programme]. (P4)

Although many participants could not find the necessary information, others had already begun the process of recognition as RN several years ago, only to have it interrupted when the Norwegian Directorate of Health needed to update the requirements. This led to confusion and frustration among the participants. After the Norwegian Directorate of Health updated the requirements, the participants discovered that the courses they had previously taken were no longer applicable and that they had to start over again:

When I arrived in Norway in 2012, I went with my friends in [name of the Norwegian town] … and studied national subjects for health personnel for two months, and then, I completed my clinical period in geriatrics afterwards. When I was in the middle of completing the mental health clinical period, the rules changed. Then, the dream of becoming a nurse suddenly stopped, and it took me 11 years before I decided to start again. (P7)

After many years of waiting and when they were finally admitted to the bridging programme, some also experienced challenges related to the digital aspect of the programme. The participants had mixed feelings about digital teaching methods. Although some found it convenient and effective, others highlighted drawbacks, such as reduced engagement and limited interaction with fellow students. They appreciated the flexibility and accessibility of digital platforms but also acknowledged challenges, such as distractions at home and technical issues. Group work posed its own set of challenges, including disparities in effort and interruptions during meetings:

It [digital teaching] is not the same as if you go to school and then sit together and discuss … and then if you have any questions, you can raise your hand and receive answers to questions”. (P3)

Another participant said the following:

About advantages and disadvantages … Yes, perhaps you become a little less engaged. Because you’re at home, right? You get a little distracted by things at home. But I think it’s not that bad, really. But of course, if it is digital, you will have little contact with your fellow students. (P2)

Despite these challenges, the participants recognised the importance of effective communication and the patience of teachers in adapting to digital teaching methods. They also expressed a desire for better preparation and training in digital tools to enhance the learning experience.

The participants also shared various experiences regarding financial support and arrangements made with their employers to pursue their studies and become RNs. Although some received scholarships from trade unions or employers and were granted study leave with pay, others had to negotiate time off or rearrange their work schedules. Some managed their expenses through small student loans, while others received financial support from their employers for specific periods, such as clinical studies or exams:

I received four months’ full payment during my clinical studies in geriatrics and mental health. (P4)

Another participant experienced a few difficulties, but he managed them well:

I didn’t get leave, but I had to rearrange some shifts so that I could attend classes. Therefore, there was no issue with the work schedule. It worked out well for everything. (P8)

Despite differences in financial support received, the participants expressed gratitude for supportive managers and institution directors who facilitated their academic pursuits:

I received a bachelor’s scholarship from my employer. It was a one-time payment, but it required a commitment of two years. I received NOK 30,000, which was almost equivalent to one month’s salary. Although it wasn’t a significant amount, considering the time I was away from work, it greatly supported my studies. I only received paid leave when I had to take the exams. (P1)

### An emotional journey – the ups and downs of participating in the program

This theme reveals the students’ perspectives on participating in the programme and getting a job as a nurse. The participants expressed various emotions and reflections on their journey as healthcare workers and becoming nurses. One of the participants came to Norway in 2010 and received her authorisation as an RN in 2023, a process that was felt as like being on an emotional rollercoaster. Most of the participants expressed a sense of loss and stagnation because of the long time spent working as a healthcare worker, which they perceived as wasted:

I feel that I have already lost many years, and I feel that I have stagnated as a healthcare worker. (P4)

Another participant shared his disappointment upon initially failing an exam, but found strength and support from friends and colleagues to persevere and ultimately succeed:

I was disappointed when I did not pass the exam, but my friends and colleagues supported and encouraged me, and I did not give up and I managed to complete and pass the exam. (P5)

Another participant described feeling saddened by working as a nursing assistant for many years but acknowledged the necessity of waiting for the opportunity to pursue further education.

Looking back, most of the participants felt that they were fortunate to be given the opportunity to attend and fulfil the programme. The participants reflected on their journey with a mindset of resilience and determination, acknowledging the challenges they faced but also questioning how they managed to overcome them. One of the participants shared his reflections, as presented in the following statement:

I came to Norway with a mindset that … whatever comes, I will handle it. So, it wasn’t easy when I look back, but at that time, you also think it will be okay. But looking back, I just ask myself … ‘How did I manage it?’ (P7)

Despite the lengthy process of authorisation as RNs and the challenges they encountered along the way as they ‘navigated towards the shore’, all the participants felt gratitude to be enrolled and being able to complete the programme. The participants expressed numerous positive aspects of their experiences with the education programme. Several participants highlighted the ability to balance studies with family life and maintain dedication to their jobs without feeling like they lost anything. The availability of part-time study options was particularly appreciated by those with families, as one of the participants said:

Yes, but especially for those who have a family, it is very important to have the opportunity to attend a part-time study … So, it was very positive that we got a part-time study. Yes, I am very grateful for that. (P6)

The participants found the experience highly inspiring, often feeling emotional during their time at the programme and bonding with peers over shared experiences. The participants expressed immense gratitude for the opportunity provided by the education programme. One of them said the following:

It was very much inspiring. I was almost in tears or had goosebumps many times on the first day [at the programme]. We share the same experience … You have no idea how grateful we are for the opportunity we have been given through [programme] … (P7)

The participants also recommended the educators for their support and passion, emphasising the confidence they instilled in them to continue and follow the programme despite challenges. Achieving self-fulfilment despite challenges was noted by another participant in the following statement:

For me, it was self-fulfilment. Finally, I have achieved something here in Norway, despite the challenges … (P8)

### Achieving recognition – the journey to authorisation and professional confidence

This theme expresses students’ sense of ease upon their former education being finally acknowledged and becoming a nurse. After attending the programme and finally receiving their authorisations as RNs, all the participants suddenly felt confident, independent and responsible. The participants gladly shared their experiences and perspectives on their new roles and responsibilities as nurses.

Some of the participants highlighted the differences between being a healthcare worker and a nurse, noting the heavier physical work and administrative tasks in the former role, even though it was mentally more exhausting because of the need to consider various aspects of patient care. One of them said the following:

There are more administrative tasks. There are many dosages, medicine and administration. I think it’s much easier. But it is more mentally exhausting. You must think about everything. You must take care of all things. (P1)

One participant expressed happiness in his new position, attributing it to the healthcare environment, colleagues and professional challenges. They acknowledged the increased responsibility and developmental opportunities in their new roles. One of them said the following:

Yes, I’m really enjoying the new role now. I feel that there is a lot of responsibility, but at the same time, it is very … What can one say, developing? I feel that I do much more than I have done in the past [as a healthcare worker]. It has something to do with responsibility. (P7)

Other participants found enjoyment in daily nursing tasks and embraced the increased responsibility that came with their new role despite the busier workload. One said the following:

I enjoy it because I get to do nursing tasks every day. I dose medicine and take blood tests … doctor’s visit and such. I have great fun with such new challenges. And new experiences. But I have got more responsibility in any case … (P2)

The participants expressed confidence in transitioning to the role of a nurse, noting that their previous experience as healthcare workers facilitated their familiarity with routines and administrative tasks. Several participants mentioned that, despite their previous roles as healthcare workers, they were occasionally called upon to perform nursing tasks, such as inserting injections, thus having the opportunity to demonstrate their skills. One of the participants said the following:

… But when I was a healthcare worker, they would sometimes pick me up to insert an injection. Because they know I’m fully qualified, if there were no nurses on the ward … (P4)

Overall, the participants expressed gratitude that their previous working experience and knowledge from both the Philippines, East Africa and the Norwegian healthcare system contributed to their readiness for the role as nurses, ultimately leading to their well-deserved authorisation as RNs.

## Discussion

The aim of the present study was to gain knowledge about the experiences of students in DIGISKOMP-D, related to being a nurse educated outside the EU/EEA and a student with a Norwegian as a second language (N2) and migrant/refugee background.

Data analysis revealed that the participants were committed to the long-term goal of becoming RNs in Norway, a process that typically spanned from seven to thirteen years starting from their arrival in Norway until they obtained Norwegian authorisation as RNs. Despite facing numerous challenges, the participants exhibited hope, passion and perseverance in their pursuit of receiving authorisation. Rather than succumbing to difficulties or abandoning their dreams and returning home, because of their high resilience, they persevered and overcame challenges. This is in accordance with results from a literature review conducted by a Norwegian research team [29] that described IENs’ individual resilience, contextual protective factors and structural protective factors as contributing factors to overcoming challenges. None of the participants considered quitting after entering the programme, and none lost their hope that, one day, they would become RNs in Norway. These findings can be associated with the Brazilian theorist and pedagogue Paulo Freire’s [42] message that ‘there is no change without dreams, as there is no dream without hope’ (p. 81), thus suggesting that hope is an approach to education that enables the process for agency to flourish.

Previous studies [43, 44] have demonstrated that newly arrived refugees and migrants may experience asymmetric power relations, powerlessness and a lack of dialogue when interacting with authorities. Similar findings have been uncovered in the present study. Specifically, all the participants felt powerlessness and experienced a lack of dialogue with the Norwegian authorities during the process of recognising their former nursing education. Furthermore, major challenges were encountered when looking for and finding information about the programme and, thus, trying to find the way out of “bureaucratic challenges”. In a figurative sense, standing up for one’s own competence, which IENs bring from their home country—and not always being humble and accepting low wages and poor working conditions—is about ‘fighting the power of the oppressors’, an idea vindicated by Freire [45]. Drawing parallels to the banking model of education, which is built on the premise that the teacher possesses all knowledge while the learners are seen as passive recipients who are expected to accept information without questioning the world [45], the participants in our study felt that their questions were not answered or that they did not engage in a dialogue with the Norwegian authorities. The banking model of education is closely associated with oppression [45]. Therefore, by analogy, one could argue that the participants experienced feelings of oppression because they felt compelled to adhere to the rules and regulations imposed by Norwegian authorities on all IENs who want to practice nursing in Norway.

Some of the participants had previously started courses that were similar to those offered by the current programme. However, because of a reorganisation of the entire programme, these former courses were not recognised, and the students were required to start anew. These challenges could potentially disappoint the participants, leading them to give up. Other challenges were related to meeting prerequisites, such as proficiency in the Norwegian language, learning about the rules and regulations of the Norwegian healthcare system and completing various courses and clinical rotations culminating in the submission of a bachelor’s thesis. These requirements contrast with those for IENs from the EU who were not required to participate in language programmes or bridging programmes to enter the Norwegian labour market because the employer may assess whether IENs’ language and professional competencies are adequate for providing safe nursing care [46].

Along with these challenges, similar to results from previous studies [47–49], the participants in our study faced personal challenges that included overcoming difficulties with employment, finances, family, bereavement for leaving their country and family for a better life in Norway. According to Duckworth [50], individuals pursuing long-term goals aligned with their personal interests and passion for the subject will persist in overcoming obstacles to achieving their goals, as seen in this case with the goal of becoming an RN in Norway. Most of the participants considered themselves ‘fortunate’ to be offered a bridging programme. The participants seized the opportunity to enrol in and attend this bridging programme. All the effort- and time-consuming challenges were suddenly forgotten when achieving their life goal of becoming RNs in Norway. However, this was not possible without them responding to challenges quickly, adaptively and effectively; hence, they persevered through the set of challenges because of their passion, perseverance, flexibility and positivity [51]. These individual qualities have been coined as ‘grit’ and comprise motivation, self-control, a positive mindset and a goal directedness to accomplish long-term objectives [50].

Although all the participants were positively to attending the digitised bridging programme, the shift from physical to digital learning reduced their opportunities for social interaction in their learning journeys. This echoes the findings from an early study [52] which revealed that a lack of social engagement with peers and educators had a detrimental impact on nursing students’ psychosocial learning environment. Interestingly, many participants in our study coped with digital disruptions or personal issues by immersing themselves in working with assignments and submissions, all while remaining steadfast in their pursuit of the ultimate goal—to become RNs in Norway. This approach emerged as a common strategy for navigating the challenges they encountered.

The bridging programme, among other learning outcomes, also included promoting the advancement of critical thinking and clinical judgement. Freire [45] focused on reflection, developing critical thinking, dialogue and learning from each other. A distinctive feature in the bridging programme is the focus on getting students to reflect, and IENs often say they were not used to this from their home countries, where it was much more about the banking model of education and being served with knowledge and not asking questions. Therefore, educators engaged in educational programmes for IENs must help IENs develop critical thinking and clinical judgement, thus preparing IENs when they become RNs to provide continuity in care and qualitative good care.

As previous research has revealed [9, 11], our study shows that, despite challenges in attaining authorisation as RNs in Norway, the participants managed to adjust themselves to the nursing role and perceive themselves as being confident, independent and responsible individuals and professionals. Similar to Freire [45] who was also concerned with tolerance, autonomy, dreams, hope and the joy of life, the participants in our study exhibited feelings of contentedness often stemming from a sense of fulfillment and satisfaction with their life circumstances and personal achievements.

We can also be critical of our own teaching/pedagogy of not being sufficiently facilitating dialogue/critical reflection, hope and allowing students room for autonomy but instead being preoccupied with all learning outcomes that must be achieved.

Upon completion of the bringing programme, IENs bring a diverse range of experiences, cultural perspectives and nursing competencies to the healthcare workforce. Their integration into the nursing profession enriches Norwegian healthcare services. Furthermore, bridging programmes equip IENs with the requisite skills and knowledge to effectively navigate the complexities of the Norwegian healthcare system. As they transition into their roles as RNs with adequate mentoring, IENs contribute to the delivery of high-quality, person-centred care while promoting diversity and inclusivity within healthcare settings. Participating in a bringing programme adds comprehensive support and resources to IENs becoming RNs in Norway, thus facilitating their successful transition into the Norwegian healthcare services.

### Study limitations and strengths

This was a small qualitative study carried out with a particular cohort from Norway; therefore, the IENs’ experiences may not be wholly representative of others in a similar situation but who are in different countries. Although we were not able to recruit all participants of the DIGISKOMP-D programme, we did have a representative mix across participants’ gender, age and experiences. Another limitation may be related to the potential that the researchers could have had an ‘insider’ perspective that may have had an influence on the study; this was recognised, and a range of strategies to enhance trustworthiness were employed (such as researchers met regularly to discuss the potential research bias and the integration of a reflexive approach during data analysis and interpretation). Despite these limitations, our findings provide unique and important insights adding to the evidence base in this area of nursing education.

### Implications for complementary nursing education

The findings from this study suggest several key strategies for ensuring the success of Norwegian complementary nursing education:

#### Educational institutions

- It is crucial to incorporate IENs’ prior clinical experience and knowledge when designing a bridging program curriculum. Moreover, the institutions should incorporate social inclusion initiatives into their core activities, addressing both pre-and post-admission phases to the bringing programme.

#### Nurse educators and preceptors

- It is essential that nurse educators and preceptors create an inclusive learning environment by understanding and respecting cultural differences. This may be achieved through targeted courses and resources for those involved in training and supervising IENs during clinical placements.

#### Policymakers and authorities

- It is vital to develop policies that address language programs, provide clear information about the application process and bridging program requirements, and offer financial support tailored to the needs of IENs. Simplifying and clarifying regulations related to employment and practice for IENs can reduce bureaucratic hurdles and facilitate smoother transitions into the workforce.

## Conclusion

The present study uncovers the numerous challenges faced by IENs seeking recognition as RNs in Norway. One of the challenges they encountered was the lengthy period between their arrival in Norway and time they received their authorisation. Additionally, the challenges were linked to meeting the requirements set by the Norwegian Directorate of Health, including securing employment as healthcare workers, and demonstrating proficiency in speaking and understanding Norwegian at the B2 level. Further challenges were associated with the ambiguities surrounding the initiation of the bridging programme, financial strains, personal issues and minor technical difficulties with the digital platforms used during the programme. Despite these adversities, all the participants demonstrated remarkable hope, passion and perseverance, remaining dedicated to their long-term aspiration of becoming RNs in Norway. Upon achieving this goal, they reported feeling confident in their nursing knowledge, capable of assuming responsibility and empowered to make independent decisions.

There is limited literature detailing the benefits of bridging programmes with various teaching and learning approaches; therefore, there is a need for more educational designs that address challenges for IENs who are integrating into Norwegian healthcare services.

## Electronic supplementary material

Below is the link to the electronic supplementary material.

Supplementary Material 1

## Data Availability

The data that support the findings of this study are not openly available due to reasons of sensitivity and are available from the corresponding author upon reasonable request. Data are located in controlled access data storage at Oslo Metropolitan University.

## Footnotes

1 The language requirements are Norwegian at B2 level based on the Common European Framework of Reference for Languages (CEFR). Available at: https://rm.coe.int/chapter-5-communicative-language-competences/1680a084c3.

## Abbreviations

IENs: Internationally Educated Nurses
RNs: Registered Nurses
EU/EEA: European Union/European Economic Area
DIGISKOMP-D programme: Digitised Supplementary education for nurses educated outside the EU/EEC-Decentralised
SKOMPD: Supplementary education for nurses educated outside the EU/EEC-Decentralised
OECD: Organisation for Economic Cooperation and Development
ICN: International Council of Nurses
COREQ: Consolidated Criteria for Reporting Qualitative research
P (followed by a number): Participant (number)
